# Dietary Supplements of Shiikuwasha Extract Attenuates Osteoarthritis Progression in Meniscal/ligamentous Injury and Obese Rats

**DOI:** 10.3390/nu11061312

**Published:** 2019-06-11

**Authors:** Yu-Wen Yen, Ying-Jiun Lai, Zwe-Ling Kong

**Affiliations:** Department of Food Science, National Taiwan Ocean University, Keelung 202, Taiwan; ywyen@scentist.com (Y.-W.Y.); yjlai@scentist.com (Y.-J.L.)

**Keywords:** osteoarthritis, meniscal/ligamentous injury, high fat diet, obesity, Shiikuwasha extract, chondrocytes

## Abstract

Osteoarthritis (OA), also called degenerative joint disease, is characterized by joint cartilage loss and is strongly linked to obesity. Medicine to alleviate pain is currently the only treatment. Shiikuwasha extract (SE) has been reported to possess valuable bioactive substances exhibiting anti-inflammatory, antiobesity, and anticancer effects. Research is limited to the use of SE in the treatment of OA and obesity. We performed both anterior cruciate ligament transections and medial meniscectomies to induce OA on Sprague–Dawley rats after 11 weeks of a high fat diet followed by 9 weeks of oral SE administration (300, 600, and 1500 mg/kg). This study showed that SE treatment could reduce weight gain and joint pain. Additionally, SE significantly decreased triglycerides and total cholesterol in plasma of the S1500 group but increased high-density lipoprotein cholesterol in the plasma of the S600 group. Meanwhile, plasma levels of tumor necrosis factor alpha (TNF-α) was significantly reduced in the S1500 groups. Histopathological findings confirmed administration of SE attenuated cartilage degeneration. Immunohistochemistry examination demonstrated that caspase 3 and phospho-Janus kinase 2 (p-JAK2) expression levels on chondrocytes were downregulated by SE treatment. Our findings demonstrate that SE can alleviate OA progression by improving obesity.

## 1. Introduction

Osteoarthritis (OA) is one of the most common musculoskeletal disorders worldwide. It is characterized by cartilage damage, subchondral bone sclerosis, cysts and osteophytes (bone spurs), and synovial tissue inflammation [1]. Worldwide statistics estimate that 9.6% of men and 18.0% of women over the age of 60 years are affected by OA [2]. The prevalence of OA is increasing because of the aging of the population in both developed and developing countries and a growth in obesity, such risk factors that lead to OA [3]. Authors showed that a body mass index greater than 30 kg/m^2^ was strongly associated with knee OA in a dose-response-dependent manner; every 5-unit increase in body mass index was accompanied by a 35% increased risk of knee OA, with the extent of this relationship significantly stronger for women than men [3]. It is anticipated that the burden of OA, which needs joint replacement surgery, mostly contributed by the hip and knee will develop a primary problem for health systems globally [4]. With the increased population of obesity throughout the world, losing weight is one of the useful strategies in managing OA.

OA has long been considered a noninflammatory arthritis characterized by increased weight bearing on a particular joint, but OA is now considered an inflammatory joint disease [5]. TNF-α is associated with local chronic inflammation of the knee joints in the synovial tissue of patients with OA [6]. It is indirectly accountable for chondrocyte death and cartilage loss and can motivate inflammatory mediators, like Interleukin 6 (IL-6), Interleukin 8 (IL-8) and nitric oxide (NO), proteases, and prostaglandin E2 (PGE2) production [7]. In addition, the oxidative stress caused by reactive oxygen species was shown to increase in the serum of patients with OA [8]. Several antioxidants, including superoxide dismutase (SOD), glutathione peroxidase (GPX), and glutathione (GSH), can scavenge oxidative stress. If oxidative stress cannot be scavenged, cartilage degrades through mitochondria damage and increased lipid peroxidation [9].

Current treatments for osteoarthritis focus mainly on pain relief rather than improving the cartilage breakdown progress. Patients with osteoarthritis are purely administered with pain-relieving drugs, such as analgesic drugs [10]. So far, present OA drugs are unable to recover damage in the OA joint, and are limited to relief of OA symptoms, and are always accompanied by its high incidence of adverse effects. [11]. On the other hand, recent systematic reviews focus on the scientific proof for potential nutraceuticals and herbal medicines, which have been considered another way to manage osteoarthritis [12,13]. Hence, lessening oxidative stress and inflammation production will perhaps be helpful to OA management. Dietary polyphenols contribute a favorable response to osteoarthritis-related inflammation [14].

Shiikuwasha, also known as *Citrus depressa Hayata*, grows natively in Taiwan and Okinawa, Japan, and is used for its juice. Flavonoid compounds are the main bioactive components in citrus fruits, containing various flavanones (naringenin, hesperetin), flavone glycosides (naringin, hesperidin) and methoxylated flavones (PMFs) [15]. Nobiletin and tangeretin, the two most common PMFs, are rich in the peel of tangerines, oranges and Shiikuwasa [15,16]. Citrus PMFs play important roles in a number of biological functions, such as antioxidant, anti-inflammatory and anti-obesity [17]. Therefore, knowing that Shiikuwasha possessed valuable ingredients, we conducted this study to further investigate the chondroprotective effects of Shiikuwasha in obese rat models.

## 2. Materials and Methods

### 2.1. Shiikuwasha Extract Preparation (SE)

The SE was provided by ARKRAY Inc. (Kyoto, Japan) and manufactured from Okinawan Shiikuwasha fruit using the reflux extraction method, as briefly described below. Shiikuwasha fruit was extracted using hydrated ethanol and then the hydrated ethanol was removed. Then the extract was filtrated to remove precipitates and mixed with γ-cyclodextrin to improve its solubility. Because SE is insoluble in water, γ-cyclodextrin was added to improve the solubility of it. Finally, the mixture dried as a powder. γ-cyclodextrin does not have any bio-functional effects and is easily excreted in the urine after parenteral administration [18]. According to the information from ARKRAY Inc., the major constituents of SE were γ-cyclodextrin (50.0%), nobiletin (8.5%), tangeretin (4.1%), other polyphenols (about 11.0%) and other ingredients (about 26.0%) [19].

### 2.2. Experimental Design and Shiikuwasha Extract Administration

**Figure 1:**
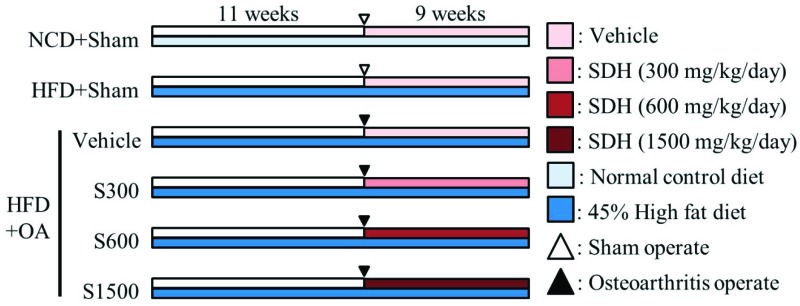
The experimental scheme used to induce obese and osteoarthritis, followed by administration of Shiikuwasha Extract.

Male Sprague-Dawley rats (5 weeks old, from BioLASCO Taiwan Co., Ltd., Taipei, Taiwan) were housed in chambers with a 12 h light and dark cycle at a temperature of 24 ± 1 °C and 40–60% humidity. A chow-fed diet (LabDiet^®^ 5001 Rodent Diet, PMI Nutrition International, Saint Louis, MO, USA, composed of 13.38% kcal from fat, 57.95% kcal from carbohydrates, and 28.67% kcal from protein) and water were consumed ad libitum as a standard diet. After one week of acclimatization, the rats were classified into six groups of six rats. Five test groups were fed a diet composed of 45.00% kcal from fat, 36.04% kcal from carbohydrates, and 18.97% kcal from protein, thus constituting the high-fat diet (HFD) groups, and the remaining group was fed a chow-fed diet containing 13.38% fat, thus constituting the normal control diet (NCD) group from week 1 to week 20. In week 12, four HFD groups underwent anterior cruciate ligament transection and medial meniscectomy (ACLT/MMx). The four groups were administered vehicle, 1X SE (300 mg/kg), 2X SE (600 mg/kg), or 5X SE (1500 mg/kg) daily for nine weeks starting 1 day after the operation. The whole experimental scheme was shown in Figure 1. Body weights were measured weekly from week 1 to week 20, and the widths of the knee joints were measured weekly by calipers (Aesculap, Center Valley, PA, USA) from week 13 to week 20. Additionally, the weight-bearing levels of the two hind limbs were measured weekly from week 14 to week 20. The animals were euthanized with CO_2_ at week 20. Immediately, blood was drawn into a syringe containing heparin and centrifuged for 30 min at 10,000 ×*g* at 4 °C. The collected supernatant was stored at −80 °C until used in assays.

### 2.3. ACLT/MMx

After anesthetization with Zoletil 50 (0.1 mL/100 g) (Virbac, Carros, France), each rat was shaved, and small incisions were cut in the medial aspect of the joint capsule (above the medial collateral ligament) to expose the right knee joint. Then, a scalpel was used to make the ACL transection, and surgical scissors were used to remove the medial meniscus [20]. The joint surface was cleaned with a sterile saline solution, and the capsule was sutured using 4-0 chromic catgut (Unik, Taipei, Taiwan). Skin was sutured using nylon 3-0 braided silk (Unik, Taipei, Taiwan). Finally, cephalosporin antibiotic (10 mg/kg) (Taiwan Biotech, Taoyuan, Taiwan) was administered subcutaneously to prevent postoperative infection. In sham animals, the joint was opened but the ACL/Meniscus was not touched and subsequently the wound was closed.

### 2.4. Incapacitance Test (Weight-Bearing Test)

Weight-bearing distribution between the postoperative and normal hind limbs was determined using an incapacitance meter (Linton Instrumentation, Palgrave, UK). The rats were stationary in a container on an inclined plane (65° from horizontal) with each rear limb placed on a separate force plate. Then, the device measured the weight of the two hind limbs independently. A total of three to five measurements were recorded and averaged for each rat. The data were expressed as the difference between right and left limbs.

### 2.5. Histopathological Analysis of Joints

After nine weeks of SE administration, the rats were euthanized with CO_2_, and the knee joints were dissected and fixed in brown vials with 4% paraformaldehyde. Tissue trimming, fixation, decalcification, paraffin embedding, sectioning, hematoxylin and eosin (H&E) staining and immunohistochemistry staining were outsourced to Rapid Science Co., Ltd. (Taichung, Taiwan). Fast green/Safranin-O and toluidine blue staining were applied to observe proteoglycan loss. The severity of the articular cartilage damage was evaluated by histopathology scoring and the Toxicological Pathology Lab (Graduate Institute of Veterinary Pathology, National Chung Hsing University, Taiwan) was commissioned to grade histopathology scores according to the standard method [21]. The evaluated items were cellular necrosis, osteophyte, loss of cartilage, subchondral sclerosis and fibrocartilaginous formation. The degree of lesions was graded from one to five depending on severity: 1 = minimal (<1%); 2 = slight (1–25%); 3 = moderate (26–50%); 4 = moderate/severe (51–75%); 5 = severe/high (76–100%). Images of immunohistochemistry were analyzed by ImageJ software (National Institutes of Health, Bethesda, MD, USA), and the percentages of positive cell were used to measure the level of protein. At least three sections from each specimen were used to determine the percentages of positive cell.

### 2.6. Lipid Profile, Liver Function, Kidney Function, and TNF-α Measurement

Plasma levels of triglycerides (TG), total cholesterol (TC), high-density lipoprotein cholesterol (HDL-C), urea, creatinine, aspartate aminotransferase (AST), alanine transaminase (ALT) and superoxide dismutase (SOD) were determined using commercial assay kits (Randox, Antrim, UK; Fortress Diagnostics, Antrim, UK) according to the manufacturers’ protocols. The plasma TNF-α and leptin level was determined using a commercial enzyme-linked immunosorbent assay (ELISA) kit (Elabscience, Houston, TX, USA) according to the manufacturer’s protocol. The intra-assay coefficients of variations were 6.21% for TNF-α and 6% for leptin; the inter-assay coefficients of variations were 5.09% for TNF-α and 6% for leptin.

### 2.7. Lipid Peroxidation Analysis

The plasma was used to measure malondialdehyde (MDA) content as described by Placer et al. [22]. In a test tube, 200 μL of reagent (15% (*w*/*v*) trichloroacetic acid and 0.25 N hydrochloric acid and 0.375% (*w*/*v*) thiobarbituric acid) were added to 100 μL plasma. This mixture was vortexed thoroughly, heated for 15 min at 100 °C, and cooled in ice for 15 min. After adding 300 μL n-butanol and mixing vigorously, we centrifuged this mixture for 5 min at 1500 × g and used the supernatant to measure absorbance at 532 nm. Using 5 nM 1, 1, 3, 3-Tetramethoxypropane as the standard, we obtained the concentration of MDA in plasma.

### 2.8. Determination of Antioxidant Enzymes

GSH content was evaluated as described by Sedlak and Lindsay [23]. The plasma was used for measurement through a reaction with 8 mg/mL 5, 5′-Dithiobis (2-nitrobenzoic acid) (DTNB) dissolved in ethanol. Absorbance was detected spectrophotometrically at 412 nm.

### 2.9. Statistical Analyses

All values are presented as mean ± standard error of measurement (SEM). Body weight, weight-bearing difference and knee width difference were evaluated through two-way analysis of variance (Two way ANOVA) followed by Dunnett’s test. Others were evaluated through One-way ANOVA followed by Duncan’s multiple range tests, and *p* < 0.05 was considered statistically significant. SPSS software (SPSS Inc., Chicago, IL, USA) was used for calculation.

## 3. Results

### 3.1. Effect of SE on Reduction of Body Weight and Fat Pad

**Figure 2:**
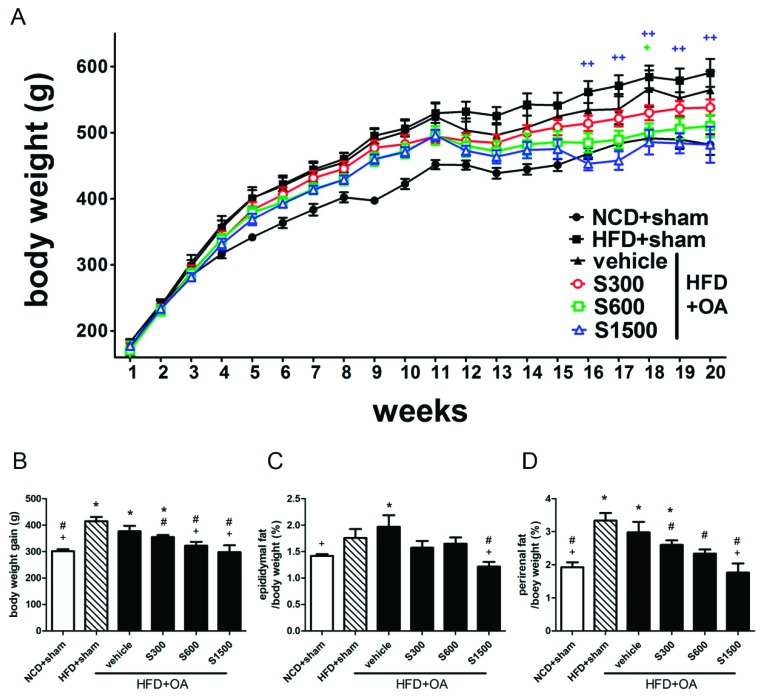
Effects of Shiikuwasha Extract (SE) on body weight and fat pad weight. (**A**) Body weight during the experiment, (**B**) body weight gain obtained from the difference between the body weights at the 1st week and 20th week, (**C**) epididymal fat weight, and (**D**) perirenal fat weight in the NCD+Sham, HFD+Sham, vehicle, S300, S600, and S1500 groups. Values are expressed as means ± S.E.M (*n* = 6). + *p* < 0.05 and ++ *p* < 0.01 when compared to the vehicle group using two-way ANOVA and Dunnett’s multiple comparisons test in Figure 2A. * *p* < 0.05 when compared to the NCD+sham group; # *p* < 0.05 when compared to the HFD+sham group; + *p* < 0.05 when compared to the vehicle group. The letters were calculated by one-way ANOVA and Duncan’s multiple range test in Figure 2B–D.

Figure 2A summarizes the effect of SE on body weight changes. From the 16th week, the rats of S1500 group began to show a slower growth compared with the vehicle group. At the 20th week (Figure 2B), the body weight of rats in the vehicle group gained an increase of 376.5 ± 20.78 g more than that at the 1st week, while that of rats in S600 and S1500 groups were significantly less than the vehicle group (322.2 ± 14.59 g and 298 ± 25.52 g in S600 and S1500 group, respectively, *p* < 0.05). Additionally, the percentages of epididymal fat and perirenal fat in body weight significantly decreased in the S1500 group (*p* < 0.05; Figure 2C,D). The administration of SE inhibited HFD-induced weight gain in rats that underwent the ACLT/MMx; higher doses were especially effective. 

**Figure 3:**
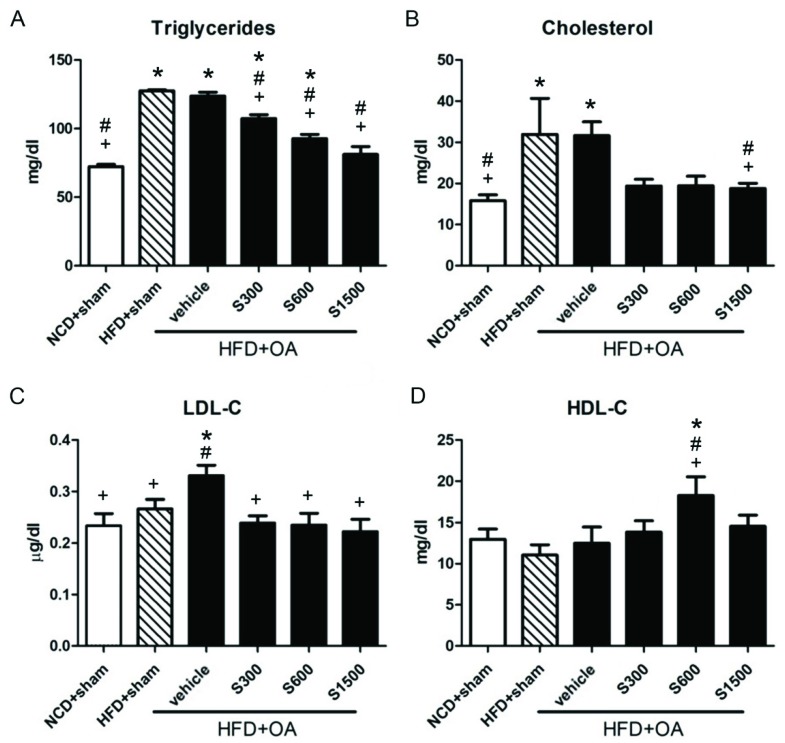
Effects of Shiikuwasha Extract (SE) on lipid profiles. (**A**) TG, (**B**) TC, (**C**) LDL-C, (**D**) HDL-C in the NCD+Sham, HFD+Sham, vehicle, S300, S600, and S1500 groups. Values are expressed as means ± SEM (*n* = 6). * *p* < 0.05 when compared to the NCD+sham group; # *p* < 0.05 when compared to the HFD+sham group; + *p* < 0.05 when compared to the vehicle group. The letters were calculated by one-way ANOVA and Duncan’s multiple range test.

Figure 3 shows that the plasma TG and TC levels significantly increased in the HFD+sham and vehicle groups compared with the NCD+sham group (*p* < 0.05). The plasma TG and LDL-C level significantly reduced in the S300, S600, and S1500 groups compared with the vehicle group (*p* < 0.05; Figure 3A,C), but the TC level was not significantly decreased in the S300 and S600 groups (*p* ≥ 0.05; Figure 3B). There was a significant difference in the HDL-C levels among the vehicle and S600 groups (*p* < 0.05; Figure 3D). The ALT, AST, urea, and creatinine levels did not show any changes among the six groups (*p* ≥ 0.05; Table 1).

### 3.2. Effect of Shiikuwasha Extract on Antioxidative Properties and Anti-Inflammatory

**Figure 4:**
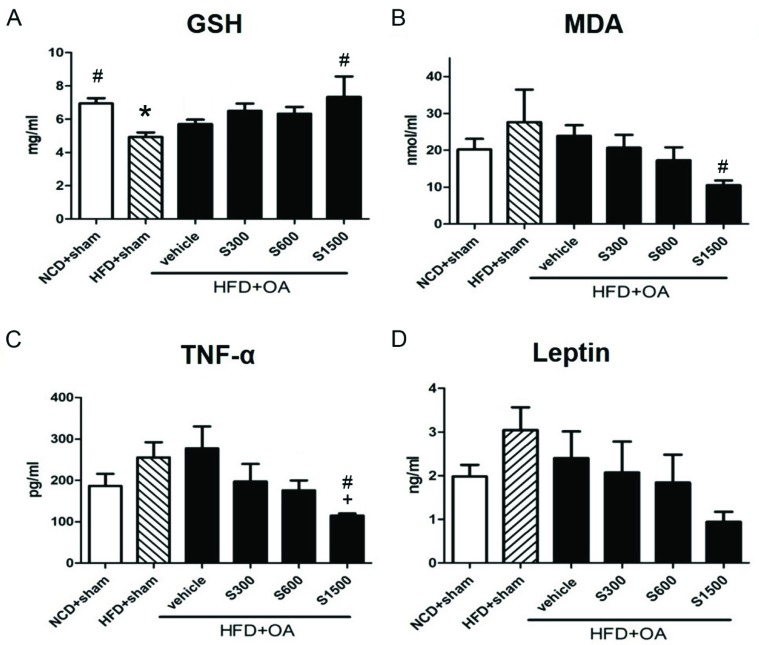
Effects of SE on GSH, SOD, MDA, leptin and TNF-α in liver and plasma. Plasma levels of (**A**) GSH, (**B**) SOD, (**C**) MDA, (**D**) leptin and (**E**) TNF-α in all groups. The data are expressed as means ± S.E.M (*n* = 6). * *p* < 0.05 when compared to the NCD+sham group; # *p* < 0.05 when compared to the HFD+sham group; + *p* < 0.05 when compared to the vehicle group. The letters were calculated by one-way ANOVA and Duncan’s multiple range test.

The plasma GSH level was increased in the S300, S500 and S1500 groups compared with the vehicle group, but without significant difference (*p* < 0.05; Figure 4A). Plasma MDA and leptin levels decreased in the S300, S600, and S1500 groups compared with the vehicle group, but without significant difference (*p* < 0.05; Figure 4B,D). The plasma TNF-α level significantly decreased in the S1500 group compared with the vehicle (*p* < 0.05; Figure 4C).

### 3.3. Effect of Shiikuwasha Extract on Attenuating Osteoarthritis Caused Pain and Damage

**Figure 5:**
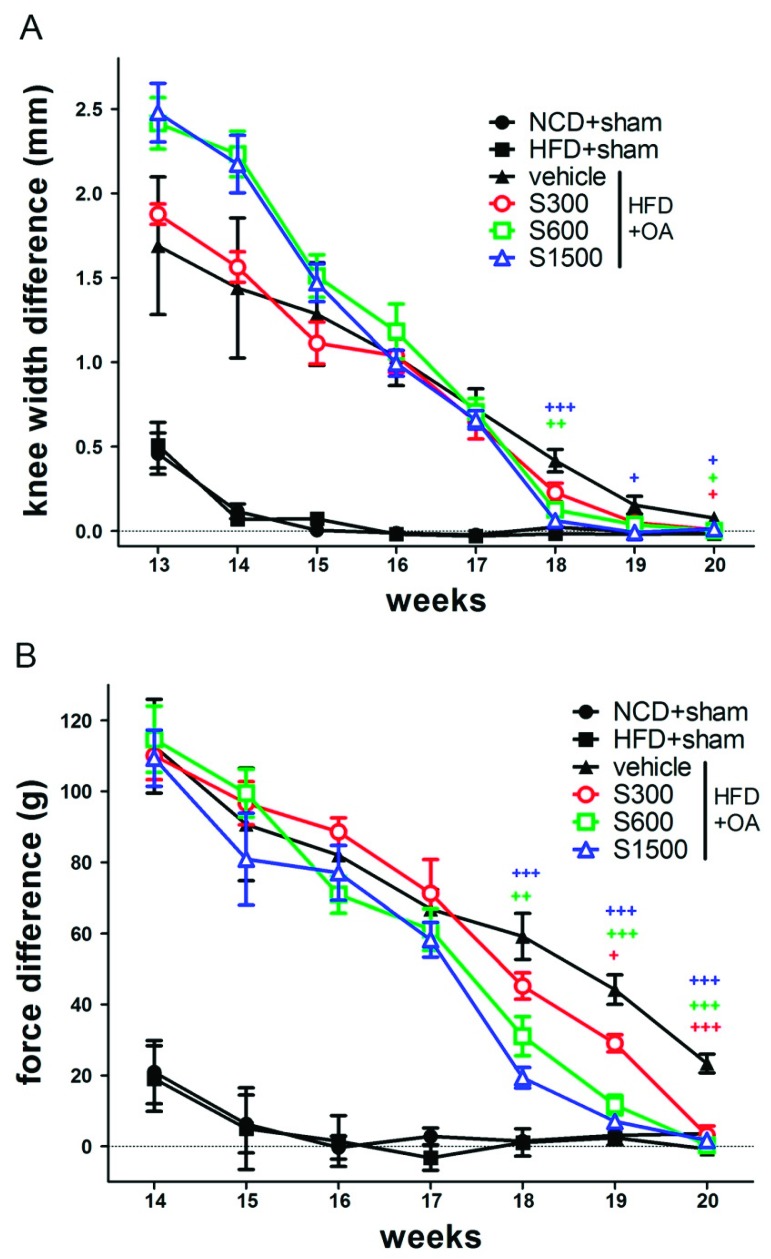
Effects of Shiikuwasha Extract on knee width and incapacitance after ACLT/MMx. (**A**) The width of the knee joint was measured weekly for eight weeks with calipers starting in week 13. (**B**) An incapacitance test was used to measure the weight bearing of the hind limb weekly for 7 weeks starting in week 14. Data were the difference between the weights applied to the contralateral and ipsilateral limbs, expressed as the mean ± SEM (*n* = 6). Two-way ANOVA and Dunnett’s multiple comparisons test were used to analyze the data. + *p* < 0.05, ++ *p* < 0.01, +++ *p* < 0.001, when compared to the vehicle group.

As shown in Figure 5A, knee swelling was not induced in the sham group, but was induced in the OA groups. At week 13, knee swelling was the most severe in the S600 and S1500 groups. At the end of the experiment, the knee width difference decreased almost to 0 mm in all OA groups. The pain induced by OA ACLT/MMx surgery changed the weight-bearing distribution in the hind legs of rats. The difference between the foot-ground reaction force placed on the right and left limbs increased after ACLT/MMx in the OA groups (Figure 5B). In contrast, the difference did not change in the sham group. After week 17, the force difference decreased faster in the S300, S600, and S1500 groups compared with the vehicle group. At the end of the experiment, the force difference in the S300, S600, and S1500 groups was approximately equal to that of the sham group. Therefore, oral administration of SE helps alleviate the pain induced by OA, as shown by the lessening of hind limbs force differences.

**Figure 6:**
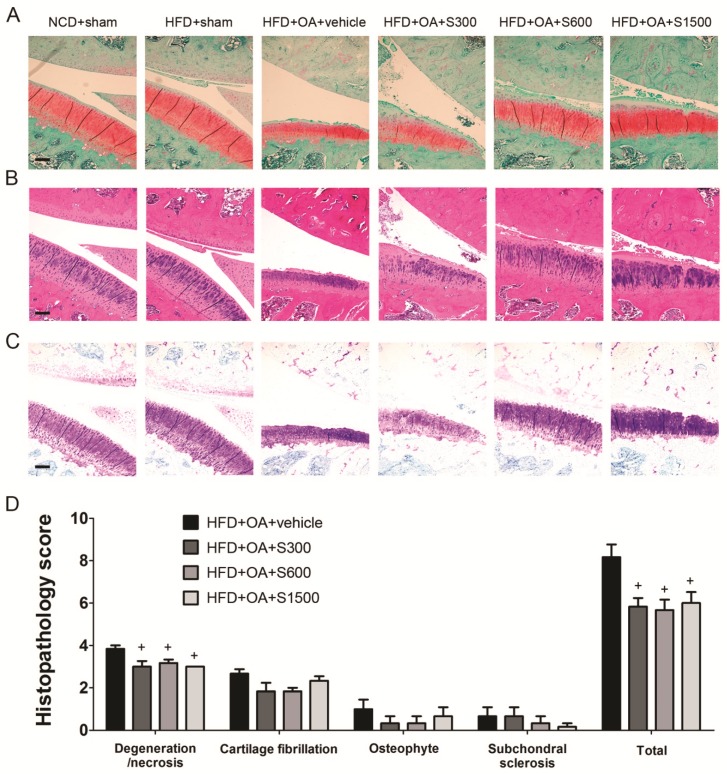
Histology examination of joints after treatment with Shiikuwasha Extract (SE). Rats underwent ACLT/MMx in the right knee. SE was administered orally daily for 9 weeks after the operation. The knee joints of rats with OA were stained with (**A**) Fast Green/Safranin-O, (**B**) H&E and (**C**) toluidine blue. (×100 magnification). Scale bars = 200 μm. (**D**) Joint sections were scored for the degree of degeneration/necrosis, cartilage fibrillation, osteophyte and subchondral sclerosis. The data are expressed as means ± SEM (*n* = 6). + *p* < 0.05 when compared to the vehicle group. The letters were calculated by one-way ANOVA and Duncan’s multiple range test.

**Figure 7:**
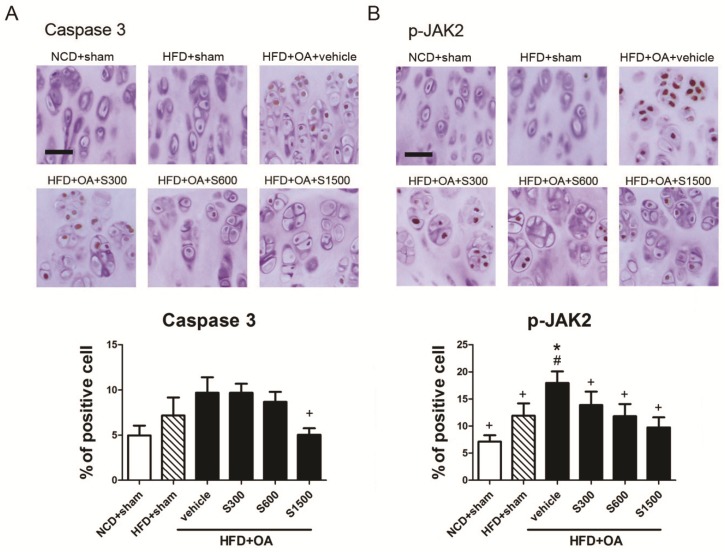
Effects of Shiikuwasha Extract (SE) on the expression of caspase 3 and p-JAK2 in osteoarthritis (OA) joints. Rats underwent ACLT/MMx in the right knee. SE was administered orally daily for 9 weeks after the operation. Immunohistochemical staining was used to identify the expression of (**A**) caspase 3, (**B**) p-JAK2 in the articular cartilage. (×200 magnification). Scale bars = 50 μm. The data are expressed as means ± SEM (*n* = 6). * *p* < 0.05 when compared to the NCD+sham group; # *p* < 0.05 when compared to the HFD+sham group; + *p* < 0.05 when compared to the vehicle group. The letters (*p* < 0.05) were calculated by one-way ANOVA and Duncan’s multiple range test.

The articular cartilage of the affected limbs in all groups was stained with Fast Green/Safranin-O (Figure 6A), H&E (Figure 6B) and toluidine blue (Figure 6C) used to observe the proteoglycan loss and morphological change. Joint sections were stained with Fast green/Safranin-O and toluidine blue to observe proteoglycan loss by OA. Treatment of SE, especially in the S1500 group, seemed likely to prevent further proteoglycan loss. The thickness of the cartilage was reduced in the subchondral bone of vehicle rats, but was improved in the subchondral bone tissue of SE-treated rats. The histopathology scores of degeneration/necrosis and total were decreased by SE treatment in S300, S600, and S1500 groups (*p* < 0.05; Figure 6D). To examine chondrocytes apoptosis, immunohistochemistry for caspase 3 were performed in rat articular cartilage. Meanwhile, immunohistochemistry for p-JAK2, which is involved in mediating the inflammatory reaction was performed. The expressions of caspase 3 and p-JAK2 were highly expressed in articular cartilage from rat with vehicle, and both were decreased by SE treatment, as assessed by immunohistochemistry (Figure 7).

## 4. Discussion

Obesity is strongly associated with OA. Excess body weight leads to wearing down of cartilage and eventually to the development of OA [24]. Body weight loss is a strategy for OA treatment due to the reduction of joint loading or mechanical force on knees [25,26]. Here we investigated the correlation between OA and obesity by using a rat model. NCD+sham and HFD+sham groups were representing healthy and obese animals respectively, as OA free control groups. Before undergoing ACLT/MMx, the rats were fed an HFD to induce obesity. In developing osteoarthritis in the knee due to an ACL injury, meniscectomy might be the most essential risk factor [27]. The ACL injury accompanied by meniscal tear has been observed in as high as 80% of osteoarthritis patients with an ACL-deficient knee [27]. To mimic real human osteoarthritis conditions, we performed ACL+MMx surgery on the rat model. In SE only dealing with the obesity aspect, SE showed anti-obesity effects in HFD-induced obese mice [28]. This study was consistent with our results that SE in the dose of 1500mg/kg could inhibit weight gain and decrease fat pad weight, TG, and TC in HFD-fed rats that underwent ACLT/MMx.

The levels of ALT and AST represented the index of liver function; the levels of urea and creatinine were represented by the index of kidney function [29]. ALT, AST, urea and creatinine did not have a significant difference among these six groups (*p* ≥ 0.05; Table 1), thus suggesting even the highest dose (1500 mg/kg) did not have the disadvantage of affecting the liver and kidney functions of rats. The following formula descripted by Nair and Jacob is used to calculate the extrapolation of 1500 mg/kg of rat doses to human doses [30]. Based on body surface area, the 1500 mg/kg dose used in the present study would equal 14.5 g/days in a 60-kg human. By this calculation, a human would have to eat 14.5 g of SE. Additionally, in a safety test, six adult males took 650 mg of powdered SE daily for four weeks and observed no adverse events (http://ebn.arkray.co.jp). Taken together, we expect that SE ameliorates the lipid profile without side effects in humans.

Physical wear and tear can cause a localized inflammatory response of the joint through increased proinflammatory mediators, such as IL-1β, IL-6, and TNF-α, in the joint space. This inflammatory response exaggerates cartilage damage through oxidative stress caused by reactive oxygen species. Cellular antioxidant enzymes are compromised in OA [31,32]. An imbalance between oxidants and antioxidants results in chondrocyte apoptosis [33]. In the present study, SE seemed likely to reverse the production of GSH and MDA that was decreased by OA-induced oxidative stress in HFD-fed rats that underwent ACLT/MMx.

The cartilage surface in rats with OA was irregular, and cartilage loss was observed. TNF-α is associated with knee cartilage loss due to a significantly higher expression of the p55 TNF-α receptor, which could cause increased susceptibility to TNF-α degradative stimuli to OA cartilage [7]. Perhaps because of decreasing TNF-α level, SE histologically improved the condition of rats with OA as evaluated by histology scoring of HFD-fed rats that underwent ACLT/MMx. Tangeretin and nobiletin suppressed the production of matrix metalloproteinase 9/progelatinase B induced by IL-1β in rabbit synovial cells in a dose-dependent manner. Nobiletin was shown to block pro-MMP-9 production and reduce its mRNA and exhibit reduced PGE2 caused by IL-1β in the synovial cells [34]. Nobiletin significantly improved the viability of human osteoarthritic chondrocytes stimulated with IL-1β and remarkably reduced the levels of nitrite, IL-6, and PGE2 through the inhibition of the JNK/ERK/p38 MAPK and PI3K/Akt pathways [35]. Combining our findings and this research indicated SE-mediated chondrocyte protection correlated with the downregulation of inflammation.

The irregular damage of cartilage existent in OA has been thought to link with chondrocytes under stress situation trends to apoptosis [36]. Therefore, caspase 3 as the key mediator of apoptosis was observed by immunohistochemistry. We found caspase 3 expression was highest in all groups, but inhibited by SE. It seemed like SE was able to improve chondrocyte death by decreasing apoptosis. The apoptosis of chondrocytes activated via the JAK2/signal transducer and activator of transcription 3 (STAT3) signaling pathway in osteoarthritis in vitro and in vivo model [37,38]. During the progression of OA, leptin as a proinflammatory regulator could trigger the JAK2/STAT3 pathway to enhance the expression of cartilage-degrading matrix metalloproteinases (MMP), including MMP-13, which are involved in OA cartilage damage [37]. Here, we also found serum levels of leptin might be decreased by SE treatment without significant differences. p-JAK2 also might be decreased by SE treatment. It could be a proof demonstrated that inhibiting apoptosis was through the JAK2-STAT3 signaling pathway.

## 5. Conclusions

In conclusion, SE can reduce body weight, leading to lighter loading on the joint. Furthermore, SE can improve blood lipid profile and cytokine TNF-α to control the inflammatory response. Additionally, SE treatment could effectively hinder swelling and pain in joints, and inhibit cartilage destruction, chondrocyte disappearance, and proteoglycan depletion possibly through the JAK2 pathway. In the future, the study of the mechanism of action could clarify the protective effect of SE against OA.

## Figures and Tables

**Table 1:** Effects of Shiikuwasha Extract on liver and kidney function.

	ALT (U/L)	AST (U/L)	Urea (mmol/L)	Creatinine (mg/dl)
NCD+sham	21.80 ± 6.05	31.75 ± 3.89	15.06 ± 0.07	0.68 ± 0.05
HFD+sham	19.56 ± 10.06	33.41 ± 3.95	14.98 ± 0.08	0.74 ± 0.02
HFD+OA+vehicle	20.92 ± 3.03	33.47 ± 4.56	15.28 ± 0.04	0.77 ± 0.01
HFD+OA+S300	21.53 ± 3.76	34.31 ± 7.01	15.03 ± 0.30	0.68 ± 0.02
HFD+OA+S600	19.03 ± 3.21	35.01 ± 7.39	15.24 ± 0.08	0.73 ± 0.03
HFD+OA+S1500	20.66 ± 6.00	31.49 ± 2.94	14.82 ± 0.53	0.72 ± 0.02

Alanine transaminase (ALT), aspartate transaminase (AST), urea, and creatinine were tested in the NCD+Sham, HFD+Sham, vehicle, S300, S600, and S150 groups. Values are expressed as means ± SEM (*n* = 6). Mean values did not differ significantly among groups calculated by one-way ANOVA and Duncan’s multiple range test.
